# Bio-inspired untethered fully soft robots in liquid actuated by induced energy gradients

**DOI:** 10.1093/nsr/nwz083

**Published:** 2019-07-11

**Authors:** Liang Xiong Lyu, Fen Li, Kang Wu, Pan Deng, Seung Hee Jeong, Zhigang Wu, Han Ding

**Affiliations:** State Key Laboratory of Digital Manufacturing Equipment and Technology, Huazhong University of Science and Technology, Wuhan 430074, China

**Keywords:** fully soft robot, untethered, induced energy gradients, Marangoni propulsion, robot swarm

## Abstract

Soft robotics with new designs, fabrication technologies and control strategies inspired by nature have been totally changing our view on robotics. To fully exploit their potential in practical applications, untethered designs are preferred in implementation. However, hindered by the limited thermal/mechanical performance of soft materials, it has been always challenging for researchers to implement untethered solutions, which generally involve rigid forms of high energy-density power sources or high energy-density processes. A number of insects in nature, such as rove beetles, can gain a burst of kinetic energy from the induced surface-energy gradient on water to return to their familiar habitats, which is generally known as Marangoni propulsion. Inspired by such a behavior, we report the agile untethered mobility of a fully soft robot in liquid based on induced energy gradients and also develop corresponding fabrication and maneuvering strategies. The robot can reach a speed of 5.5 body lengths per second, which is 7-fold more than the best reported, 0.69 (body length per second), in the previous work on untethered soft robots in liquid by far. Further controlling the robots, we demonstrate a soft-robot swarm that can approach a target simultaneously to assure a hit with high accuracy. Without employing any high energy-density power sources or processes, our robot exhibits many attractive merits, such as quietness, no mechanical wear, no thermal fatigue, invisibility and ease of robot fabrication, which may potentially impact many fields in the future.

## INTRODUCTION

Offering remarkable features that are different from conventional stiff robots, non-conventional robotics, such as soft robotics [1,2], molecular robotics [3–5], nature-inspired [6–10] and bio-hybrid robotics [11], has been changing our expectations of robots [12,13] and furthermore the design, fabrication and control strategies of robotics [14,15]. Among these, inheriting the great ability of being intrinsically deformable from soft materials, soft robots exhibit high morphological compliance and are hence adaptable to complex and unstructured environments with rather simpler designs [16–19], especially in the scenarios of co-existence of robots and humans where close collaborations are necessary [20]. Usually, to deliver enough power to drive a robot, tethered designs are widely used, since they are most convenient to implement in practice [21]. However, to exploit the full potential of such non-conventional robots, untethered designs are preferable [22]. An elegant design of a compact untethered mobile robotic system as well as corresponding fabrication and control techniques is, nevertheless, among one of the grand challenges in the general robotic community [23] yet and remains a challenging technical task when it comes to soft robotics [22].

Apart from traditional battery-powered systems [24], quite a few exciting approaches such as high-pressured gas generated by decomposing H_2_O_2_ [14], internal combustion [15,25], dielectric elastomer actuator (DEA) [10], photon-guided artificial muscle actuation [11] and magnetic actuation [26,27] have been recently demonstrated to remove the electrical or pneumatic tethers from the soft robots successfully. Although tethered wiring or tubing is eliminated, taking stiff materials and high energy-density processes off the robots, external instruments with large footprints are still necessary to support the smooth running of the robots in the last two approaches [21], consequently leading to limited performance of adaptability or accessible space. Different from the above two mechanisms, another common method is based on onboard low energy-density power sources, but it is difficult for the robot to achieve agile locomotion and miniaturization concurrently [24]. Furthermore, with high energy-density power sources adopted, designing untethered soft robots often encounters an irreconcilable conflict between the low mechanical/thermal performance of soft materials and the endless demands on high mechanical/thermal performance of high energy-density processes, especially when making a fully soft robot [14]. Hence, it is becoming a consensus to seek a simple design of an agile untethered soft robot in this nascent field.

**Figure 1.**
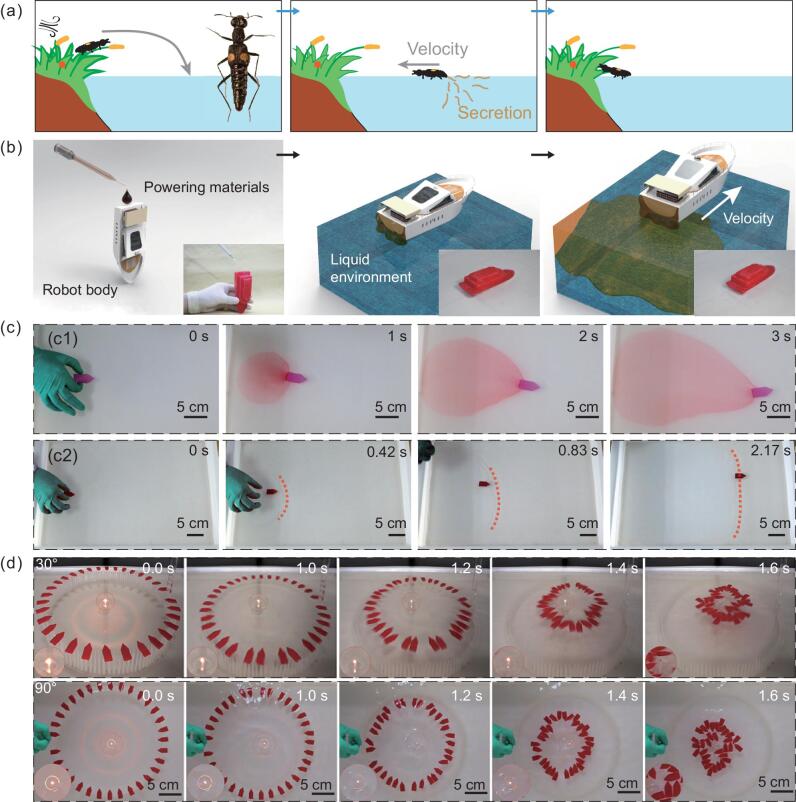
Inspiration source, schematics, motion visualization and application demonstration of untethered fully soft robots in liquid. (a) The escaping behavior of rove beetles in genus Stenus. Once the rove beetles accidentally fall on the water, they will secrete a surface-active material at their tail to gain a burst of speed to escape. (b) Schematic illustration of fulfilling an untethered fully soft robot. Deploying the powering materials on a prefabricated fully soft robot body and then contacting to the liquid environment; an untethered movement of the fully soft robot was achieved. (c) Motion visualization experiment. (c1) With the powering materials (PEG 400) and the robot body dyed red (Supplementary Fig. 2c), the movement of the powering materials and robot was clearly observed. (c2) To visualize the interaction between the powering material and liquid environment, a soft robot (Supplementary Fig. 2g) with perfluorodecyltrichlorosilane (FDTS) as the powering material was released in a water tray with a thin layer of gasoline on the water, where we can see a clear interacting line highlighted by dashed lines. (d) The robot-swarm demonstration. By aligning a robot swarm on a custom-designed circular releasing dock, 32 robots (Supplementary Fig. 2d) are released simultaneously to target a burning lamp wick immersed in kerosene on plastic foil floating in the geographical center of the releasing dock. From two perspectives, we clearly observed the directed movement of the swarm and the process of extinguishing the targeted fire flame.

‘Tears of wine’ was first observed in 1855 [28] and consequently recognized as an interfacial phenomenon of mass transport due to the surface-tension gradient [29-31] or, in brief, the Marangoni effect. The usage of a surface-tension gradient in propulsion by a few insects was discovered in succession [32-34] and then named as Marangoni propulsion consequently [35,36]. Among these insects are rove beetles in genus Stenus—a kind of beetle that generally lives around ponds and streams. By secreting and releasing surface-active materials at their hind end when falling upon water accidentally, they can obtain a propelling force resulting from the induced surface-tension gradient and push themselves to their favored terrestrial habitats (Fig. 1a) [34]. This process can be regarded as a typical energy-conversion process, in which a part of the surface energy reduced by their secretions is transformed to their kinetic energy. In our daily life, camphor/soap boats are commonly used to demonstrate such a propulsive mechanism by an asymmetric release of surfactants at the rear of the boat on water [37]. Occurring at the interface and room temperature, one of the advantages of such a propulsive process is no performance requirements imposed on the structural materials, suggesting another route to design untethered mobile robots using soft materials. Unfortunately, most work on this propulsive motion has focused on near-randomly running droplets/particles [38,39] or simple the moving of camphor boats [40,41] and few maneuver-related and application-related demonstrations [42,43] have been conducted in the past 100 years, not to say exploiting its merits from diverse perspectives.

Inspired by the escaping behavior of rove beetles, we propose here a type of untethered fully soft robot in liquid, simply based on the spontaneous release of powering materials at the interface, which will produce multiple energy gradients that include but are not limited to the surface-energy gradient to drive the robot. The actuation and maneuver of the robot can be independent from the fabrication of the robot body and other functional modules, which greatly simplifies the design of untethered fully soft robots. Compared to the existing untethered soft robots, without introducing any high energy-density power sources, it can achieve a fully soft untethered design and agile locomotive capability. It works at room temperature and does not comprise any mechanical moving parts, leading to many merits such as low-demanding material performance and no thermal/mechanical fatigue. Specifically, in this work, we intend to demonstrate an elegant strategy of designing, fabricating and maneuvering untethered fully soft robots in liquid as well as exploiting some typical potential applications.

## RESULTS AND DISCUSSION

To illustrate our approach of achieving untethered fully soft robots, we fabricated a prototype fully soft-robot body with the widely used soft silicone rubber, PDMS (polydimethylsiloxane; the detailed fabrication process can be found in Supplementary Fig. 1). By loading particular powering materials on the robot body, the robot is equipped with untethered locomotive capability in liquid. When released in the liquid environment, it is able to move agilely in an untethered manner. The robot itself features a fully soft-robot body and untethered locomotion capability. Fig. 1b thoroughly describes the procedures for actuating untethered fully soft robots of this class, containing the loading of powering materials on a prefabricated fully soft-robot body, the release of powering materials to the ambient liquid environment and the resultant movement of the robot. The key idea in the procedures is to expose particular powering materials mounted on a fully soft-robot body to the ambient liquid environment. (The ambient liquid environment in this work refers to water, unless otherwise specified.) In addition, the robot can be functionalized as well, by carrying different functional materials or components, although we do not demonstrate that here. Although the demonstrated prototype fully soft robot in this work contains no functional modules, it still has some appealing applications, one of which is demonstrated in Fig. 1d. When combined with other soft techniques such as flexible electronics and microfluidic chips, the soft robot can be enabled with more functions without any integration issues. Remarkably, the actuation and maneuver of the robot can be accomplished separately from the fabrication of the robot body and other functional components, which allows designers to focus on the functions and the robot itself to maximally fulfill the demands from specific applications, instead of compromising with the constraints from materials and corresponding fabrication and integration techniques. Supplementary Figs 2 and 3 show the custom-made soft robots and their geometry for different experiments and demonstrations produced with different materials and fabrication techniques.

According to the principle of Marangoni propulsion, surface-active materials, among which surfactants stand out for their exceptional ability of surface activation, can function as powering materials. But, beyond that, numerous other materials are found to be able to serve as the powering materials to propel the robot and will be illustrated in the subsequent context. Four representative powering materials, namely 4-Dodecylbenzenesulfonic acid (DBSA), polyethylene glycol dodecyl ether (BRIJ L4), polyethylene glycol (PEG) and silicone oil as summarized in Table 1, are used in this work to clarify our principles. The amounts of powering materials used in specific experiments and demonstrations in this work are listed in Supplementary Table 1.

**Table 1.** Physical properties of the four typical EAMs.

Materials	Water solubility	Surface tension (dyn/cm)	Features
DBSA	Soluble	26.0	Anionic surfactant
Brij L4	Soluble	32.1	Non-ionic surfactant
PEG 400	Soluble	46.6	Non-surfactant
Silicone Oil	Practically insoluble	20.8	Non-surfactant

With the help of dye, we can observe both the movement of the robot body and the molecule migration of the powering material (PEG 400) on a macro scale (Fig. 1c1). The area of dyed powering material (PEG 400) on water expands at a rather moderate speed and a clear concentration gradient below the robot always exists. In addition, with a thin layer of gasoline on the water, an apparent interaction line where the oil is pulled away by the powering material (perfluorodecyltrichlorosilane) while the robot is running is clearly observed (Fig. 1c2). Differently from the phenomenon in Fig. 1c1, the interacting line here is fast enough to outstrip the robot and slows down afterward. Furthermore, by carefully aligning 32 robots towards a burning flame on an open water surface, we demonstrate a robot swarm that can be highly centered to the same spot to assure a hit on the given target as accurately as possible to extinguish the flame (Fig. 1d and Supplementary Movie 1). The robots succeeded in putting out the fire as a swarm, under the interactions with each other and the liquid environment. Expectably, the robot swarm is able to fulfill more complex tasks in the future when combined with additional functional parts.

**Figure 2.**
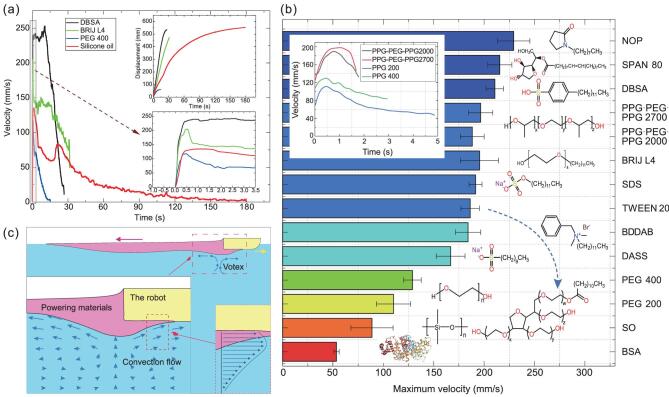
Experimental investigations of the kinetic performance of the prototype robot under diverse powering materials and the actuation mechanism underneath. (a) Full velocity profiles and corresponding moving displacement of four typical powering materials in an unlimited water environment. The data acquisition is done in a water trough (800 cm × 20 cm) in order to simulate the movement in the water environment of infinite area. The amount of the powering materials in all performance-evaluation experiments can be found in Supplementary Table 1. In addition, all the tested robots in the performance-evaluation experiments are the same as shown in Supplementary Fig. 2c. (b) Maximum velocity of various powering materials with their chemical structures. A water tray (37 cm × 53 cm, the same as the container in Fig. 3 and Supplementary Fig. 5) containing water of finite area serves as the testing environment. The inset in the upper-left corner shows the velocity profiles of the powering materials with similar molecular structures but different molecular masses. (c) Illustration of the propulsive convection flow in the liquid bulk phase induced by the powering materials. The velocity gradient near the robot is shown in the zoomed inset.

To investigate the intact kinetic characteristics of the prototype fully soft robot (Supplementary Fig. 2c, weights 0.78 g) driven by a specific amount of powering materials (Supplementary Table 1) in an infinite liquid environment, we tested the robot in a water trough (∼800 cm × 20 cm) to simulate the infinite liquid environment condition and obtained four almost complete velocity profiles of four typical substances from the very beginning to the end, respectively (Fig. 2a). All the velocity profiles show a rapid velocity increment and reach the peak during the first 0.5 s, finally approaching zero at different decreasing rates. Noticeably, there is a flat stage in the velocity curves of DBSA and Brij L4, indicating the balance of the propulsion force and resistance force at that stage. Furthermore, the profiles of DBSA and Brij L4 show relatively higher acceleration and maximum speed during the time the robot is running, while those of silicone oil and PEG 400 present much lower acceleration and maximum speed.

Besides the above four materials, we also discovered that many other materials can be used as powering materials. Detailed performance profiles at the same volume (5 μL) in a water tray (37 cm × 53 cm) together with their molecular structures are rearranged in Fig. 2b. In particular, when powering materials have very similar molecular structures, such as poly(propyleneglycol)-block-poly(ethyleneglycol)-block-poly(propyleneglycol) (PPG-PEG-PPG) and PEG, the one with the higher molecular weight usually gives a better performance, shown in the inset of Fig. 2b. Here, it is worthwhile to mention that the bovine serum albumin used here is a biological aqueous solution. Though its profile is not comparable to the other pure liquid chemicals, it gives us a hint that some natural substances are also able to serve as powering materials and drive the robot with the same strategy. In repeated performance-evaluation experiments, the prototype robot can reach a peak speed of 250 mm/s with ease under the actuation of DBSA. With a body length of 45 mm, such a velocity corresponds to 5.5 body lengths per second, which is comparable with its nature counterparts such as goldfish (6.36) and about eight times as fast as the previous reported best value (0.69) achieved by the fast-moving untethered fish actuated by DEA [10] (refer to Supplementary Table 2 for some typical speeds in body length per second of natural fishes and representative untethered soft robots in liquid).

Initially, we rely on the Marangoni propulsion theory to organize and analyse the experimental data. The net force exerted on the robot is given by

(1) \begin{equation*} {{\boldsymbol F}} = \overbrace {\int_{S}{{{{\boldsymbol T}} \cdot {{\boldsymbol n}}dS}}}^{\begin{array}{*{20}{c}} {\,Hydrodynamic}&{force} \end{array}} + \overbrace {\int_{C}{{\gamma {{\bf s}}dl}}}^{\begin{array}{*{20}{c}} {Surface}&{tension}&{force} \end{array}} \end{equation*}

where $S$ denotes the interfacial area between the robot and the liquid, $C$ the contour of $S$, namely the contact line [44]. ${{\boldsymbol T}}$ is the hydrodynamic stress

tensor, $\gamma $ the surface tension, ${{\boldsymbol n}}$ the unit normal vector of $S$ and ${{\bf s}}$ the unit vector normal to $C$ while tangential to $S$. Additionally, the surface-tension force can be rewritten as

(2) \begin{equation*} \int_{C}{{\gamma {{\bf s}}dl}} = \int_{S}{{\left[ {\gamma {{\boldsymbol n}}(\nabla \cdot {{\boldsymbol n}}) - \nabla \gamma } \right]dS}} \end{equation*}

by applying the Stokes theorem. The term $\gamma {{\boldsymbol n}}(\nabla \cdot {{\boldsymbol n}})$ represents the curvature force and $\nabla \gamma $ the surface-tension gradient. Hence, the net effect of the surface-tension force consists of the vertical supporting curvature force and horizontal propulsive force associated with the surface-tension gradient.

Traditional Marangoni propulsion theory reckons that the propulsion force comes from the surface-tension gradient. Thus, strong surface-active materials with strong surface activation ability should result in high acceleration and velocity intuitively, which is consistent with our observations, except silicone oil. Silicone oil is a strong surface-active material due to its low surface tension (∼20 dyn/cm) but exhibits much lower actuating ability compared with other similar materials. We owe this to its fast-spreading behavior, since silicone oil is both strong surface-active and insoluble in water. The fast spreading leads to the short time taken for the interacting line to outstrip the robot body so that the surface-tension gradient applied on the robot vanishes quickly, and consequently the robot gains shorter accelerating time and lower maximum velocity. As for other soluble or reactive powering materials, the behavior of the interacting line varies with the properties of materials, which is already shown in Fig. 1c.

However, there are still cases where the traditional Marangoni propulsion principle is not convincing enough. For instance, in the case of DBSA in the water trough, as revealed by the experimental acceleration, the initial stage reached 1256.43 mm/s^2^ and the corresponding propulsive force was 0.98 mN. According to the traditional Marangoni propulsion principle, the theoretic value of the propulsion force can be approximated as 0.87 mN by ${{\boldsymbol F}} = w\Delta \gamma $, where *w* is the width of the robot. Even ignoring the effect of the contact angle and unsaturation, this formula is a maximum approximation. The fact that the theoretic maximum of the propulsion force is a bit lower than the experimental result here indicates that the propulsion force does not merely originate from the surface-tension gradient.

Returning to the theory shown in Equation (1), we realized that the contributions from the hydrodynamic force were ignored. A further consideration about it told us that the term of the hydrodynamic force in Equation (1) should not be simply neglected or regarded as a resistance force; it might exhibit a propulsive effect in certain conditions. As a matter of fact, the existence of convective flow induced by a surface-energy gradient, namely Marangoni flow/convection, was discovered a long time ago, but generally overlooked in Marangoni propulsion [45]. Moreover, we believe that the chemical potential-energy gradient induced dissolving and the diffusion of soluble powering materials strengthened the Marangoni flow. Once the multiple gradients are strong enough to produce a propulsive flow in the liquid bulk phase, as shown in Fig. 2c, the robot can be dragged forward by the flow and gain a higher acceleration than the traditional approximation theory predicts. More detailed propulsion-mechanism modeling can be found in the Supplementary Data.

For the sake of simplicity, we generalize the powering materials as environmentally active materials (EAMs), denoting materials capable of generating energy gradients in the liquid environment whether to produce a surface-energy gradient at the interface (surface-tension gradient) or a chemical potential-energy gradient in the liquid bulk phase to actuate the robot. Supplementary Table 3 summarizes the EAMs involved in this work.

**Figure 3.**
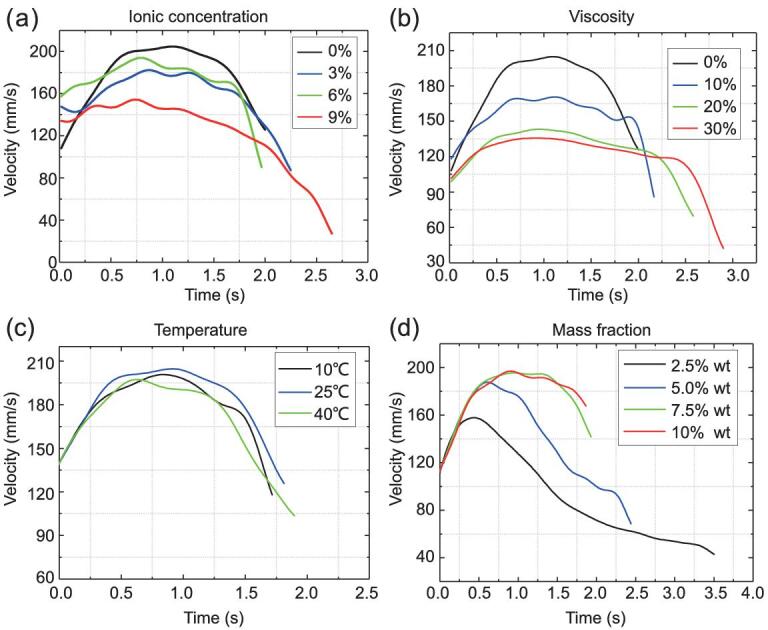
Quantitative evaluation of the kinetic performance of the prototype robot affected by various environmental factors. (a) The velocity profile of Brij L4 in water with various ionic concentrations. The ionic concentration is controlled by adding different amounts of NaCl to the water. (b) The velocity profile of Brij L4 with different viscosities of the liquid environment. The viscosity is adjusted by mixing water with different amounts of glycerol. (c) The velocity profile of Brij L4 in the water at different temperatures. (d) The velocity profile of SDS aqueous solution under different mass fractions. The larger the mass fraction, the higher the peak velocity curve. Moreover, an obvious velocity stage occurs when the mass fraction exceeds a critical value (∼5%), indicating the temporary balance of the actuation force and resistance.

Further, to understand the influence from the environments such as ionic centration (Supplementary Movie 2), viscosity and ambient temperature, the velocity profiles of Brij L4 are rearranged in Fig. 3a–c. More experimental results can be found in Supplementary Fig. 5. As expected, higher ionic concentration weakens the surface absorption, dissolving and diffusion capability of Brij L4 and hence lowers the energy gradients existing at the interface and in the liquid bulk phase, resulting in the lower velocity curve. Higher viscosity increases the overall friction in liquid and at the interface, and leads to weaker convection flow and higher resistance force, lowering velocity profiles. Meanwhile, temperature does not show any significant impact on velocity profiles in the range of 10∼40°C, which indicates the relative stability of propulsion force versus temperature and is favorable for practical applications. Apart from the liquid-state EAMs, solid ones, such as sodium dodecyl sulfate (SDS) aqueous solution, with various mass fractions can also be used to drive the robot (Fig. 3d). This suggests we have more opportunities to select a powering material in practice when facing environmental constraints. Moreover, being intrinsically soft, all these EAMs are naturally compatible with soft-robot design.

**Figure 4.**
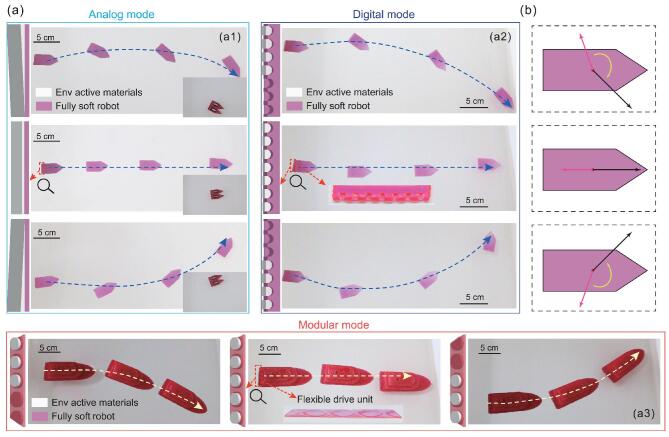
Maneuvering strategies of the robot. (a) Demonstration of three maneuvering approaches from an analog way to the digital and modular ways. In each maneuvering strategy, the custom robot is demonstrated to move linearly and curvilinearly (turning right, going straight, turning left). In each case, the spatial distribution of the powering materials is shown on the left. The soft robots for demonstration are shown in Supplementary Fig. 2c, e and i, respectively. (a1) The analog maneuvering approach. The spatial distribution of powering materials in the analog mode is continuous. The inset is the loading demonstration, where a combined robot made of two foam-based robots with a payload of 20 g was maneuvered by the same analog control strategy. (a2) The digital maneuvering approach. By deploying the powering materials discretely, the digital control approach is achieved. (a3) The modular maneuvering approach. The modular maneuvering approach is achieved by simply gluing the flexible drive unit to the rear of the robot body. The flexible drive unit is a flexible patch with powering materials loaded. The insets in (a2) and (a3) are zoomed pictures of the robot's tail, where the powering materials are mounted. (b) Force-vector analysis. The red arrow represents the resisting force, the black represents the net propulsive force and the yellow represents the turning torque.

By spatially controlling the deployment of EAMs on the robot, three maneuver modes (analog, digital, modular) are developed to control the movement of the robot in the designed direction (Fig. 4a and Supplementary Movie 3). The difference between analog and digital modes lies in the distribution form of the deployed EAMs. It is continuous in the analog mode, while discrete in the digital mode. Whether in analog or digital mode, a curved moving path of the robot can be achieved by varying the distribution of EAMs from symmetry to asymmetry. The inset in

Fig. 4a1 further shows that a combined robot made of two foam-based robots (self-weight each, 0.901 g, Supplementary Fig. 2h), each carrying a 10-g weight, can also be maneuvered with the same analog strategy.

The analog control strategy is an intuitive and basic way to achieve the spatial distribution of EAMs, but faces challenges such as high operational difficulty and low scalability. By altering the distribution from continuous to discrete, the analog mode can be digitalized. This can make the robot maneuver more precise and scalable. With a series of simple punched active-material-dispensing nozzles/reservoirs, a more precise digital maneuver of the robot can be achieved in the future. As for the modular maneuver mode, it is a significant enhancement on the digital mode at the system level owing to the natural compatibility with the modularity of the actuating approach. As mentioned before, the maneuver of the robot can be achieved independently from the fabrication of the robot body and function realization. Therefore, a flexible drive unit can be designed and fabricated separately by loading the designed amount of particular EAMs on a flexible patch at the designed location and glued to the robot that needs actuation and maneuver capability in liquid. As shown in Fig. 4a3, we fabricated a macro-soft-robot base and a flexible drive unit separately and then attached them together using simple gluing. The same maneuver strategy works on a stiff toy boat as well, as demonstrated in

Supplementary Fig. 6. As seen in Fig. 4a, without any transmission systems involved in the robot body, only the spatial control of EAM release spots is required for maneuvering. Hence, the design protocol of such an untethered fully soft robot, as well as the subsequent fabrication and maneuver process, is greatly simplified. It may free designers from tedious robot structure and control design, and allow them to focus on developing attractive features/functions according to their specific tasks/demands.

**Figure 5.**
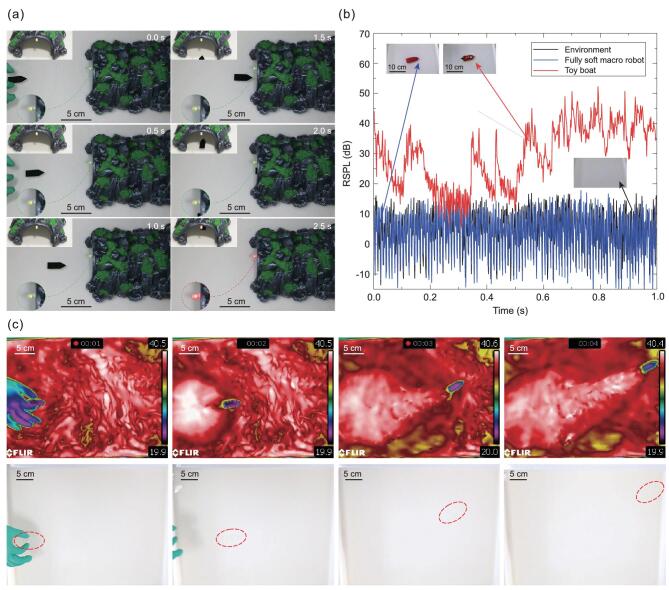
Advantageous characteristic demonstrations of the robot. (a) Photographs of a robot (black-dyed, Supplementary Fig. 2b) targeting an LED-indicated path. The targeting demonstration shows the accuracy that the robot control can achieve. (b) Comparison of the measured relative noise level of a soft macro robot and a battery-powered, similarly sized toy boat. RSPL denotes to the relative sound pressure level. The robot exhibits a much lower noise level compared to the toy boat. (c) Photographs of a cold robot (∼20°C, made from native PDMS, Supplementary Fig. 2a) moving in warm water (40°C) taken using an infrared video camera and a normal camera, respectively.

To fully exploit the potential of this type of soft robot, a variety of demonstrations have been carried out to illustrate its unique characteristics. Supplementary Fig. 7 shows that our actuation approach can work well in other liquid environments (gasoline–water interface) when a proper EAM is selected, indicating the ubiquity of our actuating method in liquids. Fig. 5a and Supplementary Movie 4 demonstrate that a back-dyed fully soft robot, following the designed path, precisely passed through a toy rockery tunnel with an optical detector underneath and triggered the lighting color change of an LED indicator. Without any redundant movement, the precisely targeting performance of the robot reveals the efficiency and accuracy of our control strategy.

By completely removing mechanical moving components and extracting the kinetic energy from the induced energy gradients in the liquid environment, our strategy settles the common issue of mechanical wear and does not introduce any additional mechanical noise during the running process, showing great quietness (Fig. 5b and Supplementary Movie 5). In addition, as unveiled by an infrared (IR) video camera, the untethered soft robot remained cold as it was in the initial state (∼20°C) when running in a warm ambient liquid (40°C), while it was almost invisible under an ordinary optical video camera (Fig. 5c and Supplementary Movie 6). This observation indicates that there is almost no heat generation inside our robot. Therefore, we have the chance to remove the damage of mechanical wear or thermal fatigue on soft structural materials and offer greater freedom to select specific structural and functional materials for attractive features. For instance, when transparent materials are used to fabricate a robot that is running in liquid at ambient temperature, an untethered robot that is invisible not only in the spectra of visible light, but also in that of IR, can be achieved using our approach. In addition, the low noise level during the operation of the robot strengthens its concealment, which makes them more appealing for prospective applications. We expect this can significantly improve the survivability of such untethered robots when approaching dangerous targets. Further, equipped with a simple and yet cost-effective fabrication process, a robot swarm can be adopted to further assure the success rate for important missions, as already shown in Fig. 1d.

In summary, we demonstrated an elegant way to design, fabricate and maneuver an untethered fully soft robot, which gained kinetic energy from the multiple energy gradients induced by the EAMs. Without the commonly adopted external or internal power sources, an agile untethered mobility of the fully soft robot with a moving speed of 5.5 body lengths per second was achieved via a mild process, and there exists almost no thermal or mechanical damage to the soft structural materials. This approach shows great promise in unlocking the potential of soft robots by enabling them with flexible maneuvering strategies and unlimited functionalization methods. For example, with a body made of dissolvable materials, such a robot carrying drugs is promising in achieving targeted drug delivery and autonomous release without any leftover. Equipped with the features of zero noise, thermal and optical transparency, the robot also has a special purpose in environmental monitoring, scout and detection. Furthermore, large-scale robot swarms can be developed affordably and organized in an advanced way to explore more untouched scenarios in the future.

## METHODS

### Soft-robot fabrication

Different kinds of soft robots were made for various experimental investigations and demonstrations. Some soft robots (Supplementary Fig. 2a–e) were made by polydimethylsiloxane (PDMS) replication. Elastosil RT601 A and B elastomers (Wacker Chemie AG Co., Germany) were mixed at a weight ratio of 9:1. The mixture was stirred for 5 minutes and placed in a vacuum desiccator for 10 minutes to dislodge bubbles. Meanwhile, a laser cutter (AOL-640, Aolei Co., China) was used to prepare a poly-methyl methacrylate (PMMA) mold (20 mm × 30 mm × 1 mm). Then, the mixture was poured into the mold and cured in an oven (UF-55, Memmert Co., Germany) at 80°C for ∼7 minutes. Silicone color dyes (Red PMS 186C or PMS BLACK, Smooth-On Inc., PA) were added to the PDMS mixture when necessary for motion visualization. However, considering the swelling effect of PDMS in gasoline, a polyvinyl chloride sheet (thickness of 0.5 mm) was used to make the robot (Supplementary Fig. 2f and g).

In the loading test, polystyrene (PS) foam was used to fabricate the robot (Supplementary Fig. 2h) via hot line cutting. Two identical foam robots were glued together (∼0.9 g) in the loading test for better stability on water. The geometrical details of the soft robots are illustrated in Supplementary Fig. 3a–d.

A macro robot (Supplementary Fig. 2i) was made by PDMS casting from a 3D printed mold by a 3D printer (Phoenix Touch Pro Translating, Full Spectrum Co., NV). The macro robot and the electric toy boat in the noise-comparison experiment were the same size.

### Environment preparation

Water was used as the main environment medium in most of the investigations. Gasoline (No. 92, Sinopec Inc.) was used as an alternative liquid environment in the experiment for Supplementary Fig. 7 and an indicator to observe the interacting line in Fig. 1c2. Glycerol (Sinopharm Chemical Reagent Co.) was used to adjust the environment viscosity. Food-grade salt, sodium chloride (NaCl), from a local grocery was used to adjust the ionic concentration of the water. Boiled water (∼100°C), room-temperature water (∼20°C) and iced water (∼0°C) from a fridge were selectively mixed to obtain the specific water temperature verified by a thermometer (TP 101, Yida Co., China).

### Robot-swarm demonstration

A segment of cotton string (Supplementary Fig. 8) that is the same as that in a candle wick was passed through circular plastic foil with a hole of a similar size to the string diameter floating on the water, where kerosene could be dispensed and attached, to serve as a burning lamp wick. A circular PMMA (*R* = 260 mm) glued with pipette tips was used as a releasing dock in Supplementary Fig. 9. With 32 robots (Supplementary Fig. 2d) aligned on the tips of the dock, it was slowly immersed into water while keeping the robot away from the water before igniting. Once lit, the releasing dock was lowered farther to release the robot swarm.

### Single robot targeting demonstration

A toy rockery tunnel (255 mm × 195 mm × 110 mm) was equipped with a green and red bi-color LED (5 mm, Wei Hengsheng, China) in the middle of its entrance. A photosensitive resistance (LXD-5 mm, Long Xinda, China) was placed under the water in the rockery tunnel (Supplementary Fig. 10a). When the black robot passed the photosensitive resistor, less light was detected so the LED switched from green to red. The process was controlled by a customized circuit (Supplementary Fig. 10b).

### Kinetic-performance measurement

A polypropylene (PP) water tray (53 cm × 37 cm × 6 cm) was filled with ∼2870 g water (14 mm in depth). To obtain an intact and realistic velocity curve, the head of the robot was kept vertical and gently put into the container. A water trough (800 cm × 20 cm × 10 cm) was used to investigate the overall movement in the unlimited liquid environment. The depth of water was also kept constant (14 mm) in the test.

### Maneuver demonstration

Prototype soft robots with and without holes at the back were filled with powering materials at different spatial locations. For the macro robot, a flexible PDMS slice with five embossments (inset of Fig. 4a3) was glued to the stern of the robot to hold the powering materials.

### Powering-materials preparation and their deployment in soft robots

All the liquid-state powering materials from the supplier (Sinopharm Chemical Reagent Co., China or Sigma Aldrich Inc., St. Louis, MO) were used without further treatment. SDS was dissolved in distilled water (25°C) in an ultrasonic agitator (KQ300DV, Kun Shan Ultrasonic Instruments Co., Ltd., China) for 20 minutes and used as an aqueous solution.

The robot was held by hand with its head down and then a tiny amount of powering material was cast onto the tail using a pipette (Eppendoff 10 μL, Germany). The robot was held for at least 10 s to achieve uniform distribution of the powering material. The volume of powering material in the experiments and demonstrations are listed in Supplementary Table 2.

### Video-tracking of the moving robots

A digital camera (EOS 70D, Canon Inc., Tokyo) with a lens (EF-S 18–135 IS STM, Canon Inc., Tokyo) was placed perpendicularly to the water surface (14 mm) to record the movement process at the rate of 60 fps until the robot struck the edge of the container. The noise test was also recorded by this camera at a distance of 75 cm from the noise source. If necessary, another camera (A6000, Sony Inc., Tokyo) parallel to the water/gasoline level was used to record the movement laterally.

The robot could stop normally before reaching the other end of the water trough, while the whole movement process was recorded in the form of picture by an industrial camera (MER-031–300GC-P, DaHeng IMAVISION Co., China) with a frame rate of 120 fps for the following data acquisition. The positions of cameras in different experiments are illustrated in Supplementary Figs 11 and 12.

### Image post-processing and data acquisition

A self-developed program based on Halcon (MVTec Software GmbH Co., Germany) was used for image post-processing. The videos were transformed into images frame by frame for subsequent processing. The velocity profiles were obtained under an image-processing algorithm as following: first, the edges of the robot at different time slots were recognized from transformed images; then, velocity and acceleration of the robot were calculated by a centroid location with a central difference scheme algorithm. Meanwhile, a filter was applied to improve the signal–noise ratio; and, finally, the centroid coordinates from the pixels were converted into real length units. More details can be found in the Supplementary Data.

## Supplementary Material

nwz083_Supplemental_Files
