# Supplementary material for: Bio-inspired untethered fully soft robots in liquid actuated by induced energy gradients
Source: Natl Sci Rev. 2019 Jul 11;6(5):970–81. doi: 10.1093/nsr/nwz083 (PMC8291417; doi:10.1093/nsr/nwz083)
Supplement: nwz083_Supplemental_Files [file nwz083_supplemental_files.zip › SUPPLEMENTARY_MATERIALS.pdf]

SUPPLEMENTARY MATERIALS

Bio-inspired untethered fully soft robots in liquid actuated by induced energy gradients

Liang Xiong Lyu[1,†], Fen Li[1,†], Kang Wu[1,†], Pan Deng[1], Seung Hee Jeong[1,⊥], Zhigang Wu[1,*] and Han Ding[1,*]

[1]State Key Laboratory of Digital Manufacturing Equipment and Technology, Huazhong University of Science and Technology, Wuhan 430074, P. R. China.

[†]These authors contributed equally to this work.

[⊥] Current address: 24 Peabody Terrace 1002, Cambridge, MA, 02138.

*Corresponding authors. Emails: zgwu@hust.edu.cn (ZG Wu) or dinghan@hust.edu.cn (H Ding).

Propulsion mechanism modeling

In a bid to model the dynamic characteristics of the robot, Newton's second law is applied:

$$\boldsymbol{F} = m\boldsymbol{a} \tag{S1}$$

$\boldsymbol{F}$ can be divided into two terms: one is the surface tension force $\boldsymbol{F}_c$, and the other is the hydrodynamic force $\boldsymbol{F}_s$ imposed on the robot by the ambient liquid flow. According to the definition of surface tension, $\boldsymbol{F}_c$ can be written as the integral of surface tension along the boundary C of liquid-robot interface S.

$$\boldsymbol{F}_c = \int_C \gamma s dl \tag{S2}$$

$\boldsymbol{F}_s$ is the surface integral of the stress on the interface S.

$$\boldsymbol{F}_s = \int_S \boldsymbol{n} \cdot \boldsymbol{T} dS \tag{S3}$$

$$\boldsymbol{F} = \boldsymbol{F}_c + \boldsymbol{F}_s \tag{S4}$$

$$\boldsymbol{a} = \frac{d\boldsymbol{x}_c}{dt} \tag{S5}$$

Substituting Eq. S2-S5 into Eq. S1, the specific form of Newton's second law of the robot is obtained.

$$\int_S \boldsymbol{n} \cdot \boldsymbol{T} dS + \int_C \gamma s dl = m\frac{d\boldsymbol{x}_c}{dt} \tag{S6}$$

Based on the Stokes theorem, $\boldsymbol{F}_c$ can be decomposed into a supporting curvaure force and a propelling force associated with the surface tension gradient, already shown in in the main text (Eq. (2)).

$\boldsymbol{F}_s$, as the hydrodynamic force exerted on the robot, also consists of two components: a supporting force associated with the pressure and a viscous force, which is obvious from the constitutive equation.

$$\boldsymbol{T} = -p\boldsymbol{I} + \mu\left[\nabla\boldsymbol{u} + (\nabla\boldsymbol{u})^T\right] \tag{S7}$$

When the liquid flow generated by the energy gradients is fast enough to drag the robot to accelerate, $\boldsymbol{F}_s$ shows a propelling behavior. We believe that the hydrodynamic force is more likely to show a propulsive effect when (i) the inertia of the robot is large and (ii) the powering material is strong surface-active and soluble. For example, when the macro soft robot (Supplementary Fig. 2i) deployed with NOP was used for maneuvering demonstration, an obvious propelling flow driven by the gradients can be observed.

The bicharacteristics of the hydrodynamic force can be regarded as a result of the velocity competition between the robot and the liquid. The robot dragged by the surface tension gradient induced force gains acceleration and velocity. Meanwhile the liquid suffering from the surface tension gradient and the chemical potential energy gradient (when the powering material adopted is soluble) also gains acceleration and velocity. Accordingly, the robot will be dragged forward or backward based on the results of velocity competition between the liquid and the robot. The larger inertia the robot possesses, the more likely the robot is to lose the game. Moreover, strong surface-active capability and solubility of powering materials avoids the fast spreading behavior like silicone oil and weak convection flow rate.

A generally propulsive convective liquid flow evolves like this: at the beginning of the robot locomotion, the chemical potential energy gradient (macro dissolving and diffusion) and the surface tension gradient (Marangoni effect) lead to a propulsive velocity gradient at the interfacial surface, which pushes the robot forward. As time goes by, the energy gradients fade away and the propulsive velocity gradient becomes resistant, shown in Supplementary Fig. 4.

In order to solve Eq. S6, Navier-Stokes equations and convection-diffusion equation should be involved to determine the complex flow rate field. Supposing that the liquid here is incompressible, the specific form of N-S equation, continuum equation, and convection-diffusion equation in such particular case can be written:

$$\frac{\partial \boldsymbol{u}}{\partial t}+(\boldsymbol{u}\cdot\nabla)\boldsymbol{u}=-\frac{1}{\rho}\nabla p+\frac{\mu}{\rho}\nabla^2\boldsymbol{u}+\boldsymbol{g} \tag{S8}$$

$$\nabla\cdot\boldsymbol{u}=0 \tag{S9}$$

$$\frac{\partial \Gamma}{\partial t}+\nabla_s \cdot (\Gamma \boldsymbol{u}_s)+\Gamma(\nabla_s \cdot \boldsymbol{n})(\boldsymbol{u}\cdot \boldsymbol{n}) = J(\Gamma, c_s)+D_s\nabla_s^2\Gamma \tag{S10}$$

The relationship between the powering materials concentration and surface tension varies with powering materials' properties. In addition, the surface tension gradient is in relation with temperature, however the temperature induced surface tension gradient is ignored here since no obvious heat effect was observed in the experiment. Assuming the linear relationship between the surface tension γ and the powering material concentration Γ, then

$$\gamma = \begin{cases} \gamma_0 - k\Gamma & \Gamma \le \Gamma_c \\ \gamma_0 - k\Gamma_c & \Gamma > \Gamma_c \end{cases} \tag{S11}$$

Boundary conditions can be obtained directly from the no-slip velocity conditions and the stress conditions between the Gas-Liquid interface.

$$\boldsymbol{u}|_S = \frac{d\boldsymbol{x}_c}{dt} \tag{S12}$$

$$\boldsymbol{n}\cdot \boldsymbol{T}|_{S'} = \gamma \boldsymbol{n}(\nabla \cdot \boldsymbol{n}) - \nabla\gamma \tag{S13}$$

Even though we have all these equations to describe the phenomenon, it can hardly be solved analytically. There are mainly two barriers to solving these partial differential equations. The first is the highly intercoupling between the equations. Secondly, some parameters in these equations are hard to measure in the experiment and modelling in theory, such as $J(\Gamma, c_s)$. The numerical solution would be a better choice to qualitatively analyze this problem since the equations are highly coupled together.

The physical meaning of the symbols involved above is listed in Supplementary Table 4.

Image processing algorithm

Each single image was transformed from RGB space to HSV space for color segmentation. The approximate location of the robot was obtained via edge detection and color recognition. In order to improve processing efficiency and accuracy, the original image was cropped to a small rectangle consisted of the robot and background around the approximate location. A canny operator was used to extract subpixel precise color edges of the robot. With the edges of the robot, the centroid was determined easily. The velocity and acceleration of the robot were calculated by the centroid location. The scale of the image was calculated by comparing the width of the robot in pixels with real length units. At the end, the centroid coordinates from the pixels were converted into real length units. After the raw data of robot position was obtained in Halcon, it was imported into the workspace of MATLAB (2016b, MathWorks Inc.) for post-processing and visualization of velocity.

During recognizing the robot contour in Halcon, a random concomitant error was often generated due to the limited image quality and environmental disturbance. As the contour of the robot was the benchmark of centroid location for velocity computation in MATLAB, this error often led to apparent variations of the corresponding waveform. Under such situations, a proper selection of filters takes on an important role in the post-processing program in MATLAB. Here, a central difference method and an appropriate butterworth IIR filter were adopted to improve the signal-noise ratio. By observing the spectrum map, we found that the main frequency of the effective signal and disturbance were located at rather a low frequency and about 20Hz respectively. Hence, high-pass butterworth filters with passband corner frequency and stopband corner frequency of 2Hz and 10Hz respectively, were used to filter the waveform in order to get a better and smoother profile (Supplementary Fig. 13). For the experiments in the long open tabular duct, a measuring tape hung over the water surface was used for position calibration. The value of each scale was obtained by optical character recognition. The real position of the robot along the length direction was computed through the comparison of the scale and the robot's centroid position. The region of the untethered fully soft robot was segmented by a gray threshold. The centroid of the fully soft robot was obtained by calculating the first

moment of area. In such cases, a mean-value filter and median-value filter were used instead of the butterworth IIR filter in the post-processing program.

In order to calculate the velocity and acceleration rate of the robot, the centroid location of the robot was chosen. Two kinds of scheme (upwind and central) of the algorithm were tested in our self-developed codes. It was proved that different algorithms have no effect on the velocity profiles in Supplementary Fig. 14. The central difference algorithm was chosen to calculate all the kinetic performance quantitatively.

Supplementary Tables

Supplementary Table 1. The volume of powering materials in the experiments and demonstrations

Scenes	Volume (µl)
Kinetic performance measurements in a water tray	5
Kinetic performance measurements in a water trough for silicone oil	2
Kinetic performance measurements in a water trough for PEG 400	2
Kinetic performance measurements in a water trough for DBSA	0.5
Kinetic performance measurements in a water trough for Brij L4	0.5
Loading demonstration	5 (each)
Motion visualization, gasoline/water interface motion, maneuver demonstration, targeting demonstration, infrared visualization	5
Noise comparison with toy boat	25
Swarm demonstration	0.5 (each)

Supplementary Table 2. Typical values of speed of natural fish and untethered soft robot in liquid

Type	Mechanism	Speed (lengths/sec)
Tuna	Creature	13.4
Gold Fish	Creature	6.36
Tiefeng Li et al, Sci. Adv, 2017.	DEA	0.69
R. K. Katzschmann et al, Sci Robot, 2018.	Pump & Hydraulic	0.5
Hyung-Jung Kim et al, Smart Materials and Structures, 2012.	SMA	0.35

Supplementary Table 3. Powering materials used in this work

Material Name	Type	CAS Number
N-Octyl pyrrolidone (NOP)	Nonionic surfactant	2687-94-7
SPAN 80	Nonionic surfactant	1338-43-8
DBSA	Anionic surfactant	121-65-3
PPG-PEG-PPG 2700	Nonionic surfactant	9003-11-6
PPG-PEG-PPG 2000	Nonionic surfactant	9003-11-6
BRIJ L4	Nonionic surfactant	9002-92-0
Sodium dodecyl sulfate (SDS)	Anionic surfactant	151-21-3
TWEEN 20	Nonionic surfactant	9005-64-5
Benzyldodecyldimethylammonium bromide (BDDAB)	Cationic surfactant	7281-4-1
1-decanesulfonic acid sodium salt (DASS)	Anionic surfactant	13419-61-9
PEG 400	Non-surfactant	25322-68-3
PEG 200	Non-surfactant	25322-68-3
Silicone oil (SO)	Non-surfactant	9006-65-9
BSA	Non-surfactant	9048-46-8

Supplementary Table 4. Nomenclatures used in propulsion mechanism modeling

Symbol	Physical significance
$\boldsymbol{F}$	The net force exerted on the robot
m	The mass of the robot
$\boldsymbol{a}$	The acceleration of the robot
$\boldsymbol{x}_c$	The position vector of the centroid of the robot
ρ	The density of the liquid substrate
Γ	The concentration of the powering material
$\boldsymbol{u}$	Velocity field of the liquid
$\boldsymbol{u}_s$	Surface velocity
∇	Gradient operator
∇_s	Surface gradient operator
J	Powering material source term
D_s	Surface diffusivity of the surfactant
c_s	Powering material concentration in the bulk
S	Robot-liquid interfacial surface
C	The closed contour of interface S
$\boldsymbol{T}$	Stress tensor of the liquid
p	Pressure field in the liquid

Supplementary Figures

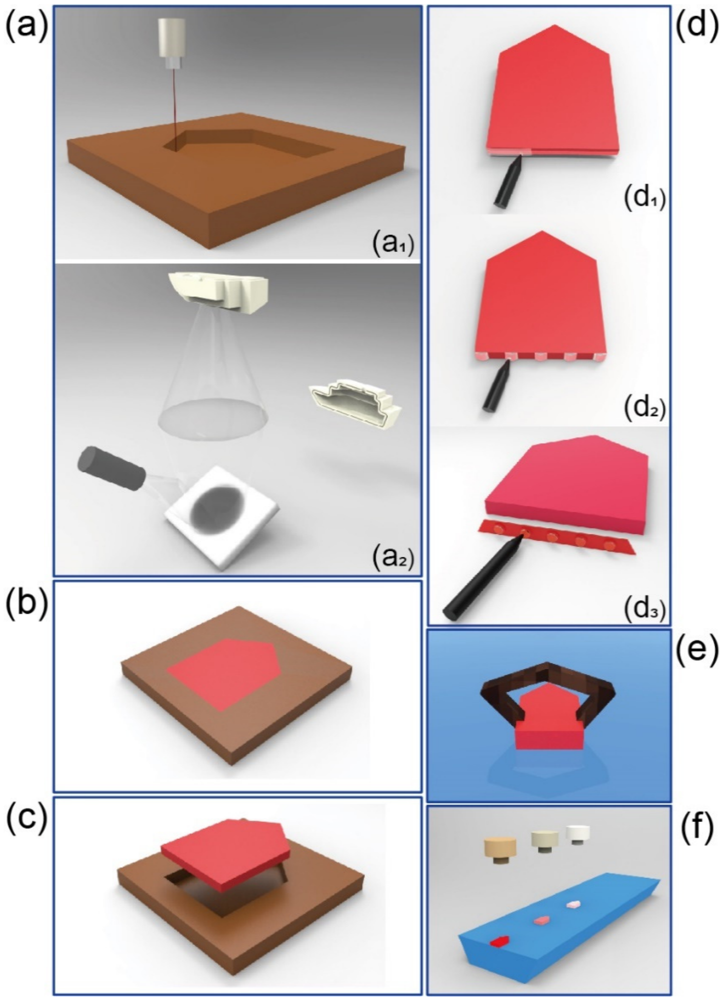

Supplementary Figure 1. Fabrication, actuation and evaluation of the untethered fully soft robots. (**a**) A mold is fabricated by laser cutting (a_1) or 3D printing technology (a_2). (**b**) Liquid PDMS mixture is poured into the mold for shaping. (**c**) After curing, the fully soft robot body is finished by removing the cured PDMS from the mold. (d) The deployment of powering materials can be done by different maneuvering approaches, such as an analog approach (d_1), a digital approach (d_2) or a modular way (d_3) to equip the robots with agile locomotive capability. Different fabrication techniques and maneuvering strategies of the robot can be combined arbitrarily when exploring different characteristics. (**e**) The releasement of the robot can be accomplished by hand or a mechanical gripper. (**f**) The performance evaluation of the robot is implemented with the assistant of video cameras and post-processing programs.

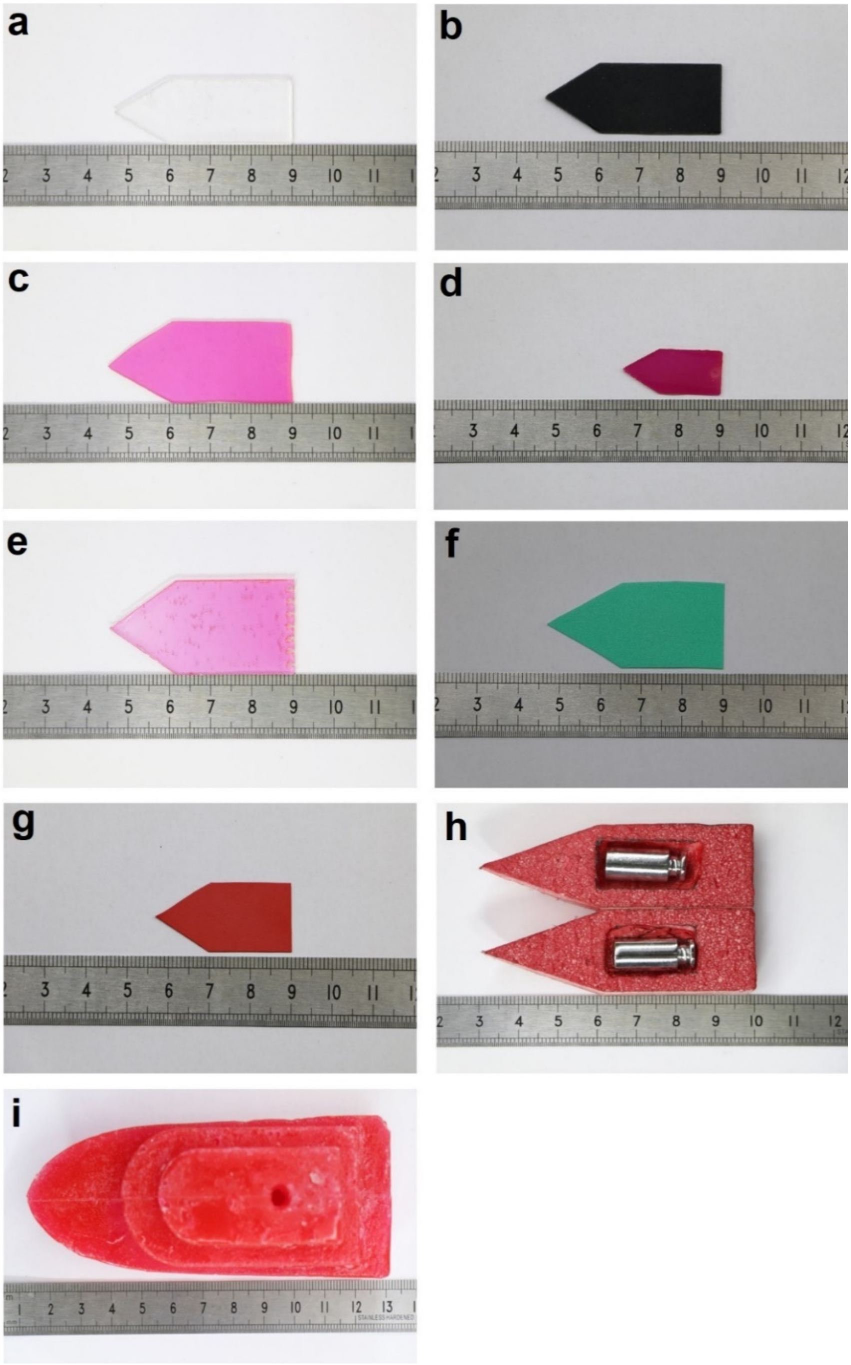

Supplementary Figure 2. Soft robots used in experiments and demonstrations. (**a**-**e**) The PDMS prototype robots with dyed ones. (**f**) The prototype soft robots made of PVC. (**h**) The combined foam robot. (**i**) The soft macro robot.

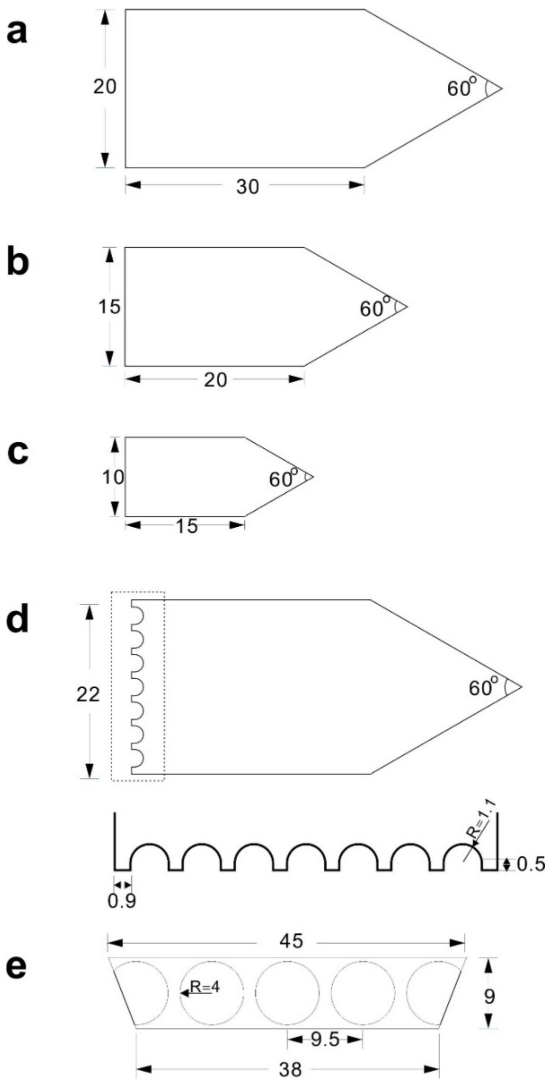

Supplementary Figure 3. The design parameters of prototype soft robots (in millimeters). (**a**) Detailed geometry of PDMS prototype soft robot and dyed ones, which were used in kinetic performance measurements. (**b**) Detailed geometry of soft robots used in motion visualization of FDTS (Fig. 1c2). (**c**) Detailed geometry of soft robots used for swarm demonstration. (**d**) Detailed geometry of soft robots used for digital maneuvering strategy demonstration. (**e**) Detailed geometry of a flexible drive unit for flexible maneuvering approach demonstration.

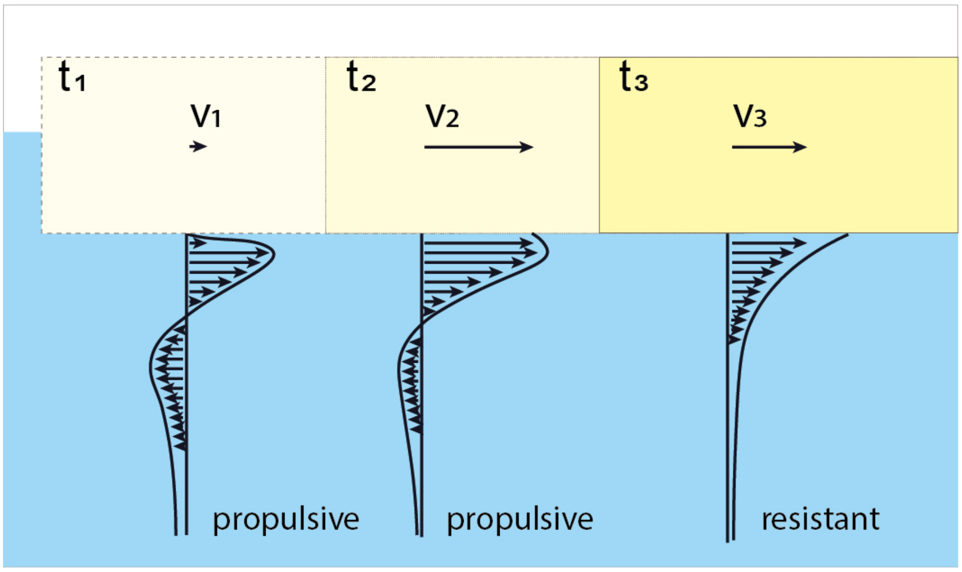

Supplementary Figure 4. Illustration of the velocity gradient in the liquid bulk phase changing versus time. In the very beginning, it's a propulsive velocity gradient near the robot according to the Newton's law for inner friction, and it turns into a resistance force soon.

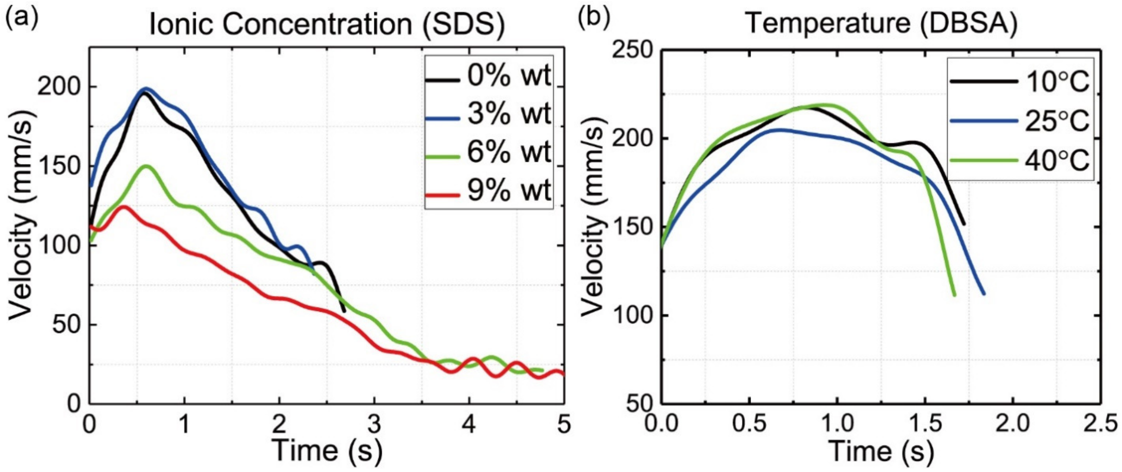

Supplementary Figure 5. Supplementary experiments of ionic concentration and temperature. (**a**) The velocity curves of the robot mounted SDS in the water with various ionic concentration. An increase of ionic concentration beyond a critical value of about 3% wt NaCl leads to the dramatical reduction of velocity of the robot. (**b**) The velocity profiles of DBSA in the water at different temperatures. The temperature change (from 10°C to 40°C) has a small effect on the velocity performance of the robot mounted DBSA.

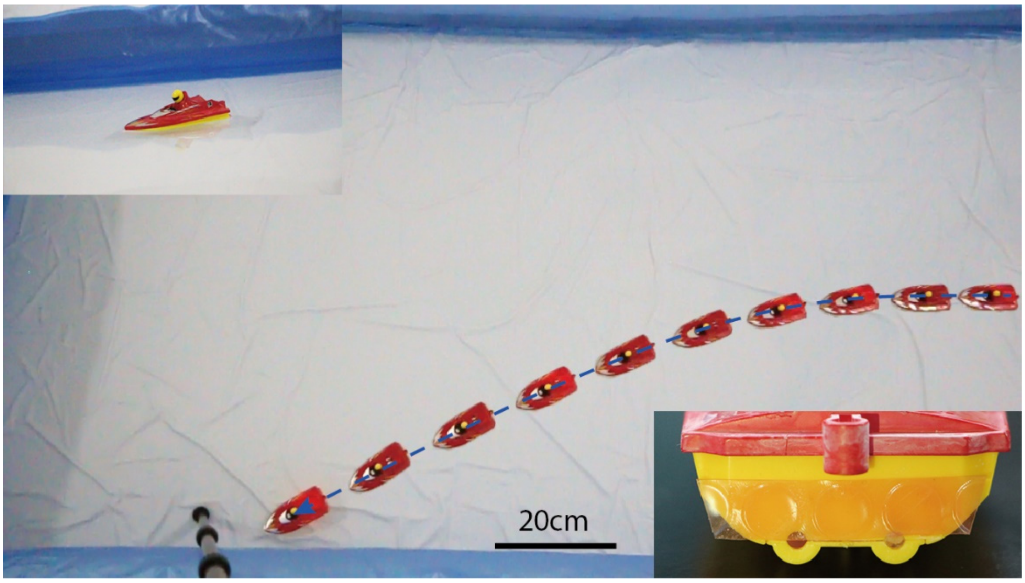

Supplementary Figure 6. Supplementary experiments of a rigid robot powered with a flexible unit in a large water environment.

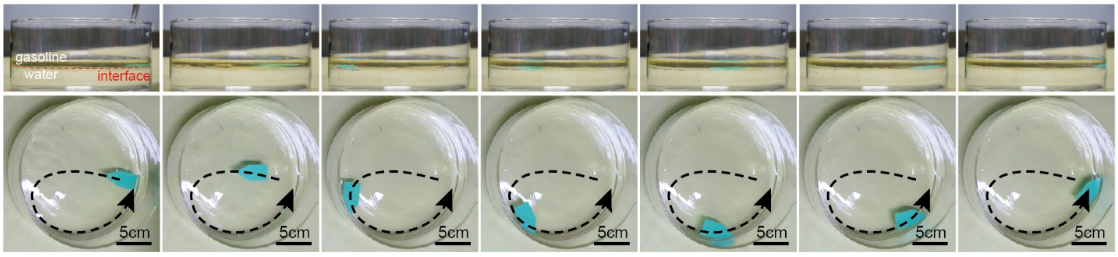

Supplementary Figure 7. Interfacial movement demonstration of the untethered fully soft robot. Driven by DBSA, the fully soft robot made of PVC is able to agilely in the gasoline-water interface, indicating the universality of our actuating method in liquids.

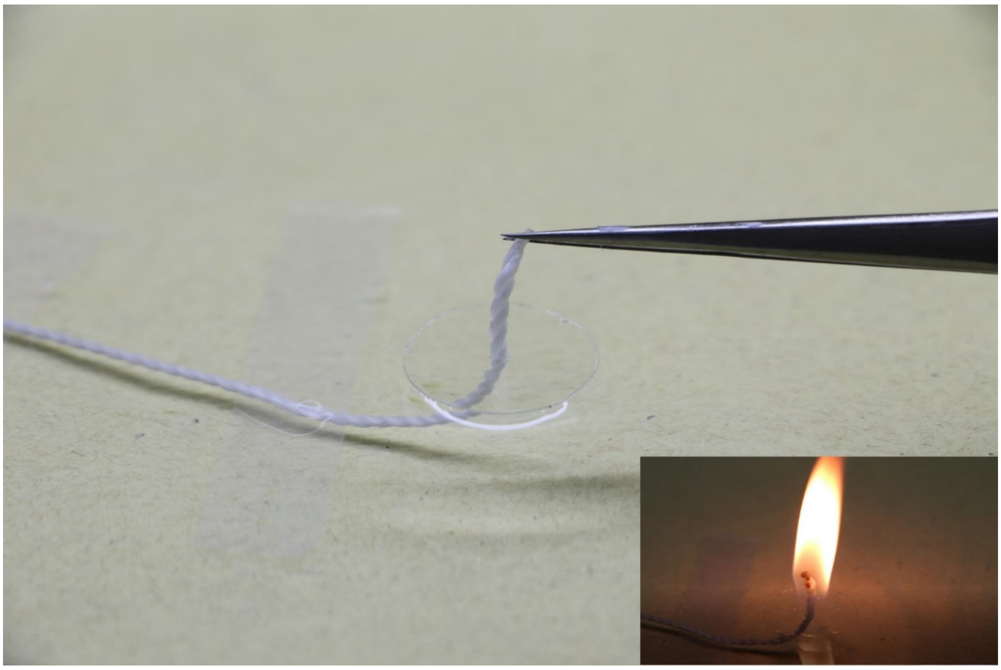

Supplementary Figure 8. The wick used in kerosene fire unlit and lit. The wick was set upright above the water and the other end was fixed at the bottom of the container with a tape. The length of burned wick is around 1 cm, so the fire will not go out before the swarm soft robots strike it.

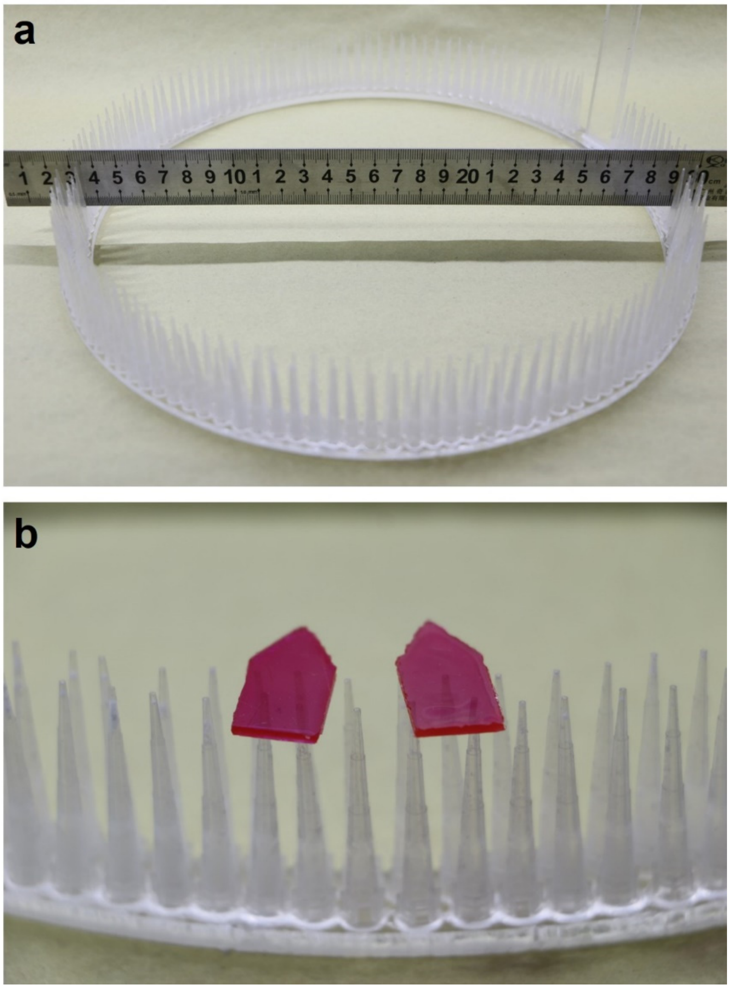

Supplementary Figure 9. The facilities for swarm demonstration. (**a**) Photo of the releasing dock. Sharp pipette tips were attached on a circular PMMA glued to support these swarm robots to assure low contacting resistance between the robots and tips. (**b**) Local details of the releasing dock with prototype soft robots.

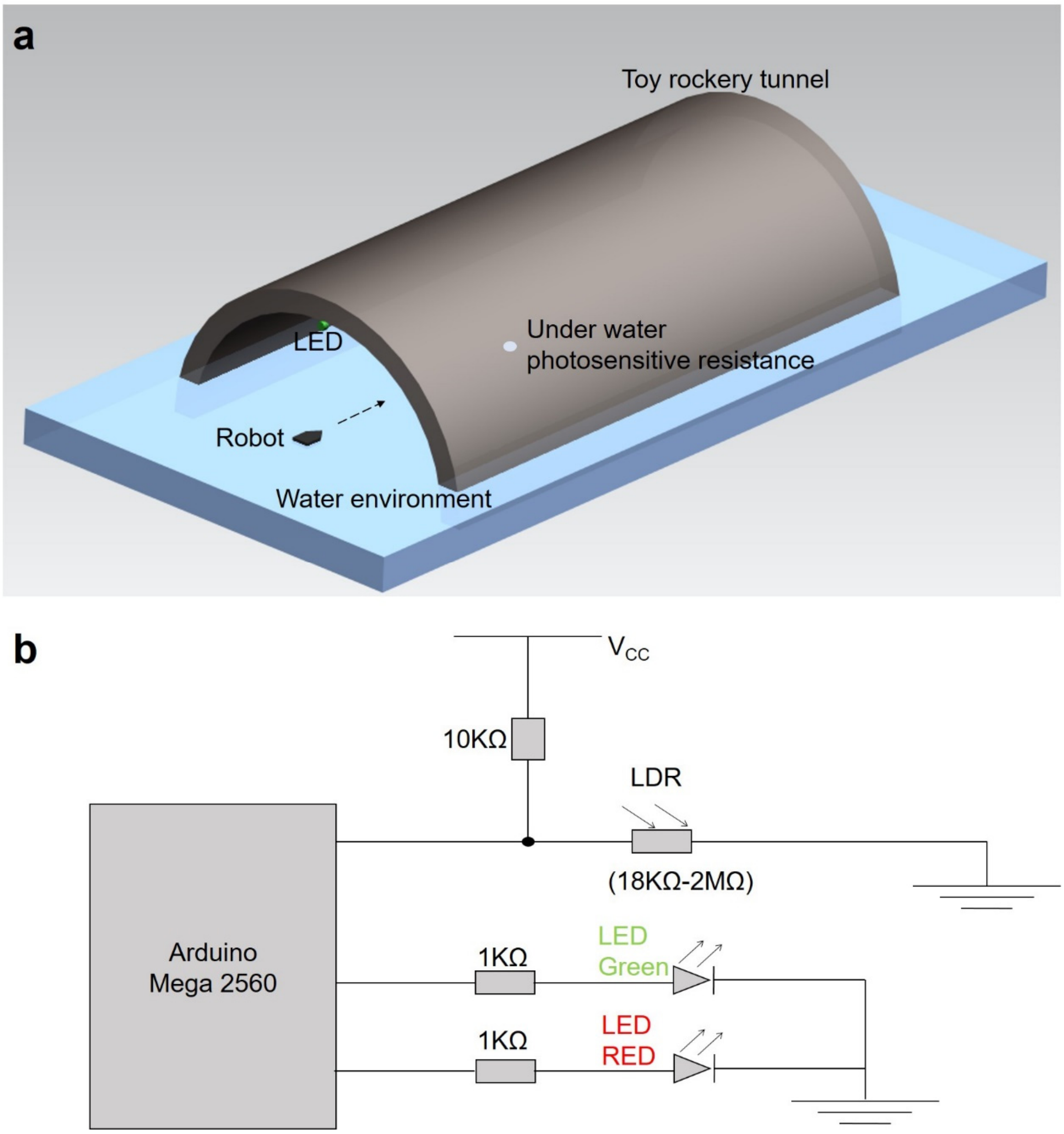

Supplementary Figure 10. The design and LED signal control system for a single robot targeting demonstration. (**a**) Scene illustration of a toy rockery tunnel placed in the water environment with a photosensitive resistance. (**b**) Control logic of the triggering and signaling circuit. When the black robot passed the photosensitive resistor, less light was detected. This action will trigger the LED by switching from green light to red light.

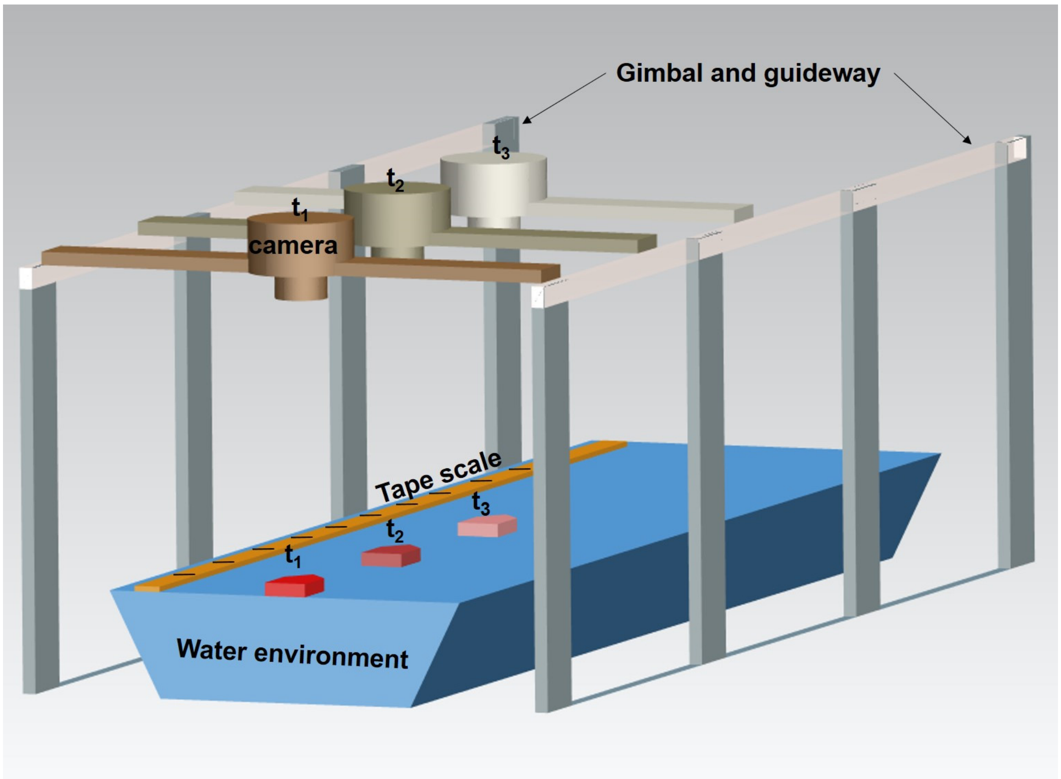

Supplementary Figure 11. Illustration of motion recording setup at different times during robot movement (t_1, t_2 and t_3) along the tabular duct. The camera was manually controlled to follow the robot's movement synchronously. The locations were determined by a tape scale along the water filled inside the tabular duct. The height of the guideway is 340 mm.

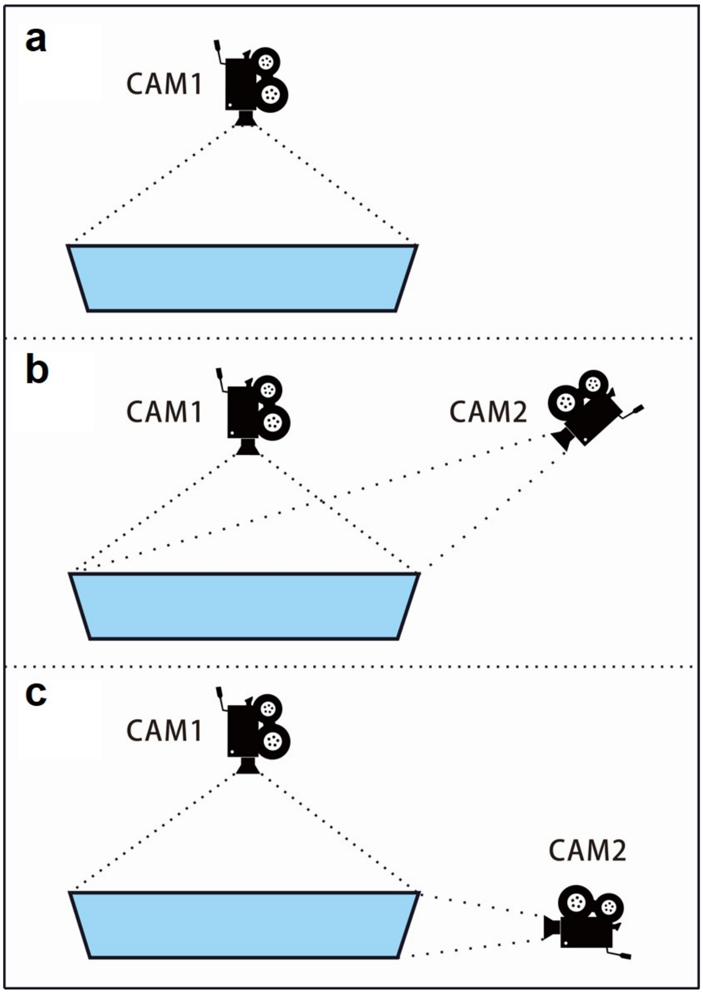

Supplementary Figure 12. The configurations of cameras in different experiments. (**a**) The position of the camera in the short water tabular container experiments. (**b**) The positions of the cameras in the robot swarm demonstration and single robot targeting demonstration. (**c**) The positions of the cameras in the water/gasoline interface test.

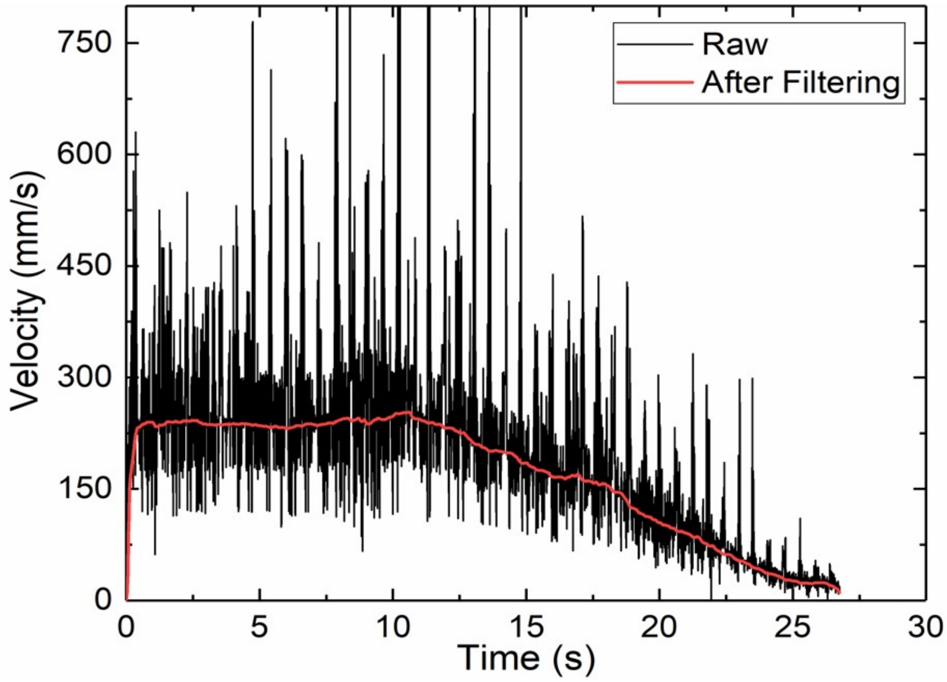

Supplementary Figure 13. Velocity profile after filtering. Velocity profile in the long open tabular duct. High-pass butterworth filters with passband corner frequency and stopband corner frequency of 2Hz and 10Hz respectively were used to filter the waveform in order to get a better and smoother profile.

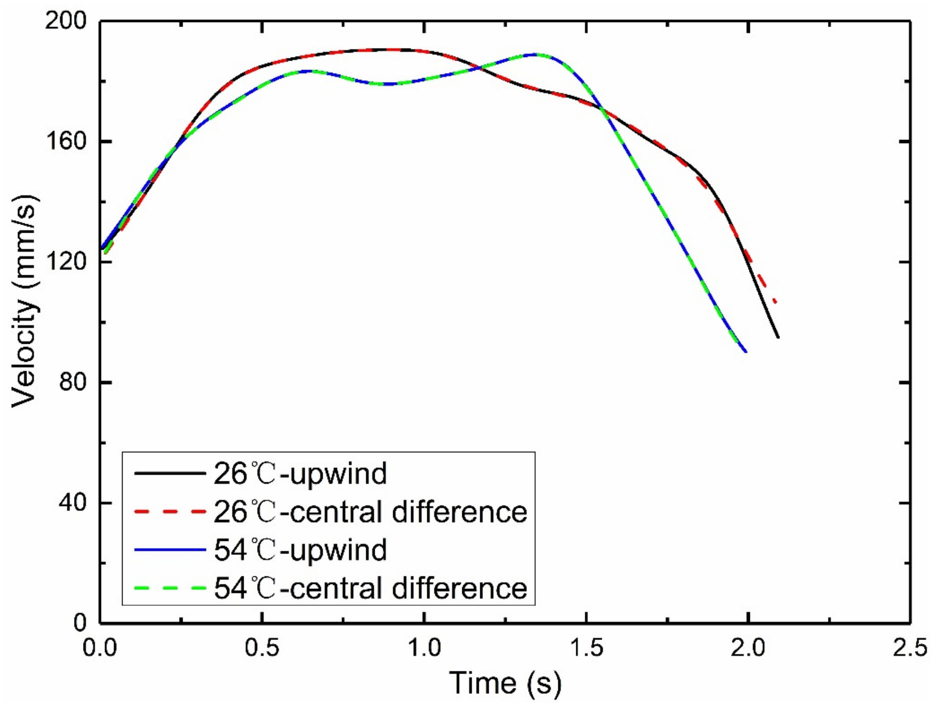

Supplementary Figure 14. Velocity profile calculated by upwind and central difference algorithms. The velocity profiles calculated by the upwind difference algorithm were plotted in solid lines, those calculated by central difference algorithm were plotted in dashed lines. Two temperature experimental conditions were chosen to prove that different algorithms have no effect on the velocity profiles.
